# Serum creatine kinase and creatinine in adult spinal muscular atrophy under nusinersen treatment

**DOI:** 10.1002/acn3.51340

**Published:** 2021-03-31

**Authors:** Maren Freigang, Claudia D. Wurster, Tim Hagenacker, Benjamin Stolte, Markus Weiler, Christoph Kamm, Olivia Schreiber‐Katz, Alma Osmanovic, Susanne Petri, Alexander Kowski, Thomas Meyer, Jan C. Koch, Isabell Cordts, Marcus Deschauer, Paul Lingor, Elisa Aust, Daniel Petzold, Albert C. Ludolph, Björn Falkenburger, Andreas Hermann, René Günther

**Affiliations:** ^1^ Department of Neurology Universitätsklinikum Carl Gustav Carus Technische Universität Dresden Dresden Germany; ^2^ Department of Neurology Ulm University Ulm Germany; ^3^ Department of Neurology University Hospital Essen Essen Germany; ^4^ Department of Neurology Heidelberg University Hospital Heidelberg Germany; ^5^ Department of Neurology University of Rostock Rostock Germany; ^6^ Department of Neurology Hannover Medical School Hannover Germany; ^7^ Department of Neurology Charité – Universitätsmedizin Berlin Berlin Germany; ^8^ Department of Neurology University Medicine Göttingen Göttingen Germany; ^9^ Department of Neurology Klinikum Rechts der Isar Technical University of Munich Munich Germany; ^10^ German Center for Neurodegenerative Diseases (DZNE) Ulm Ulm Germany; ^11^ German Center for Neurodegenerative Diseases (DZNE) Dresden Dresden Germany; ^12^ Department of Neurology Translational Neurodegeneration Section “Albrecht‐Kossel” University Medical Center Rostock University of Rostock Rostock Germany; ^13^ German Center for Neurodegenerative Diseases (DZNE) Rostock/Greifswald Rostock Germany

## Abstract

**Objective:**

To determine whether serum creatine kinase activity (CK) and serum creatinine concentration (Crn) are prognostic and predictive biomarkers for disease severity, disease progression, and nusinersen treatment effects in adult patients with 5q‐associated spinal muscular atrophy (SMA).

**Methods:**

Within this retrospective, multicenter observational study in 206 adult patients with SMA, we determined clinical subtypes (SMA types, ambulatory ability) and repeatedly measured CK and Crn and examined disease severity scores (Hammersmith Functional Motor Scale Expanded, Revised Upper Limb Module, and revised Amyotrophic Lateral Sclerosis Functional Rating Scale). Patients were followed under nusinersen treatment for 18 months.

**Results:**

CK and Crn differed between clinical subtypes and correlated strongly with disease severity scores (e.g., for Hammersmith Functional Motor Scale Expanded: (CK) *ρ* = 0.786/ (Crn) *ρ* = 0.558). During the 18 months of nusinersen treatment, CK decreased (∆CK = −17.56%, *p* < 0.0001), whereas Crn slightly increased (∆Crn = +4.75%, *p* < 0.05).

**Interpretation:**

Serum creatine kinase activity and serum creatinine concentration reflect disease severity of spinal muscular atrophy and are promising biomarkers to assess patients with spinal muscular atrophy during disease course and to predict treatment response. The decrease of creatine kinase activity, combined with the tendency of creatinine concentration to increase during nusinersen treatment, suggests reduced muscle mass wasting with improved muscle energy metabolism.

## Introduction

5q‐associated spinal muscular atrophy (SMA) is a rare monogenic lower motor neuron disease caused by mutations in the telomeric survival of motor neuron 1 (*SMN1*) gene leading to insufficient SMN protein levels. Lack of SMN protein predominantly leads to degeneration of lower motor neurons with consecutive progressive muscle wasting.^1^ Clinical subtypes are defined based on the age of symptom onset and the highest motor milestone achieved. Patients with SMA type 1 never are able to sit, while patients with SMA type 2 learn to sit without support but never are able to walk without assistance. Patients with SMA type 3 learn to stand and walk independently but may lose this ability over time.^2^ Based on exceptional study results, the United States Food and Drug Administration (FDA) in 2016 and the European Medicines Agency (EMA) in 2017 approved the antisense oligonucleotide nusinersen (SPINRAZA^®^, Cambridge, Massachusetts, USA; nusinersen) as the first disease‐modifying drug for SMA regardless of patient’s age, type, or disease stage, although these studies were designed only for interpretation of treatment effects in children with SMA type 1 and type 2 (ENDEAR^3^ and CHERISH^4^). Therefore, it is indispensable to investigate real‐world evidence of treatment effects, especially in adult patients with long‐term disease. Recently published observational studies showed promising motor score changes during nusinersen treatment.^5^, ^6^, ^7^, ^8^, ^9^, ^10^, ^11^ However, objective biomarkers are required to advise patients for treatment options and to monitor disease progression or response to treatment.^12^, ^13^ Clinical scores alone do not provide sufficient detail for this purpose. Both creatine kinase and creatinine are part of the muscle energy metabolism and are affected in neuromuscular diseases^14^ (e.g., amyotrophic lateral sclerosis (ALS)^15^, ^16^, ^17^, ^18^, ^19^ or spinal and bulbar muscular atrophy^20^, ^21^) as they reflect muscle mass and muscle integrity. Recently, decreasing serum creatinine concentration (Crn) was suggested as a biomarker for progressive denervation in SMA, thus reflecting disease progression.^22^ Serum creatine kinase activity (CK) was shown to be a promising marker for disease severity in SMA.^23^, ^24^ The aim of this study was to evaluate CK and Crn as biomarkers in adult SMA and to investigate their dynamics during nusinersen treatment.

## Methods

### Standard protocol approvals, registrations, and patient consents

Two hundred and thirty‐four patients were assessed for eligibility for this study between July 2017 and February 2020. After exclusion of patients younger than 18 years and patients with SMA type 1 (*n* = 13) or 4 (*n* = 1), as only few datasets were available, 206 adult patients with genetically confirmed 5q‐associated SMA from nine German neurological specialist care centers (Departments of Neurology in Dresden, Ulm, Essen, Heidelberg, Rostock, Hannover, Berlin, Göttingen, and TU München) were included retrospectively.

The local ethics committees of all participating sites approved the study and all patients signed written informed consent. The demographic features and clinical data of patients were collected including age, gender, baseline weight, clinical subtype, number of *SMN2* copies if available, ambulatory status, and presence of spondylodesis. Patients received nusinersen treatment by intrathecal administration according to the prescribing information for up to 18 months. Concurrently, laboratory diagnostics were performed at six time points (baseline, 2 months, 6 months, 10 months, 14 months, and 18 months), and already established motor scores (Hammersmith Functional Motor Scale Expanded ‐ HFMSE, Revised Upper Limb Module – RULM) as well as the revised ALS‐Functional Rating Scale (ALSFRS‐R) were assessed at each visit. Motor scores comprise several items rating different motor skills with higher scores indicating better function. Raters who conducted HFMSE and RULM were trained by experienced physiotherapists from the SMArtCARE initiative (Freiburg, Germany; www.smartcare.de) and ratings were performed according to the manuals. In addition, ambulatory patients performed 6‐minute walk test (6MWT) or maximum walking distance. Hand grip strength of the dominant hand was measured by KERN^®^ hand grip dynamometer MAP 80K1S (KERN & SOHN GmbH, Balingen‐Frommern, Germany) prior to start of the nusinersen treatment. Electrophysiological measurement was conducted as described before^25^ using Synergy software on Nicolet EDX^®^ System (natus^®^, Pleasanton, CA, USA) determining the motor unit number index (MUNIX), motor unit size index (MUSIX), and compound muscle action potential (CMAP) of the abductor pollicis brevis (APB) muscle of the dominant hand as a baseline assessment. Serum samples were analyzed for CK and Crn at the certified in‐house laboratory departments of each participating center using Jaffe method or comparable^26^ enzymatic reactions.

### Statistical analysis

Statistical analysis and figure drawing were performed using SPSS Statistics 27 (IBM, Chicago (IL), USA) and GraphPad Prism 5 (GraphPad Software Inc., San Diego (CA), USA). If not stated elsewhere, the assessed scores, CK, and Crn data are presented as mean ± standard deviation (SD) with the related range. As CK and Crn were not normally distributed tested by Shapiro‐Wilk test, we applied rank‐based, non‐parametric tests for the baseline analysis. To investigate the expressiveness of CK and Crn values, we correlated the baseline values with demographic features and clinical assessments using Spearman’s rank correlation coefficient (*ρ)*. We corrected for sex, weight, and height using partial rank correlation, because these variables were suspected to be confounding factors due to their influence on CK and Crn. A correlation coefficient of *ρ* < 0.3 was considered as a weak, *ρ* = 0.3–0.59 as a moderate, and *ρ* > 0.6 as a strong correlation (modified from^27^). In addition, we correlated CK and Crn with electrophysiological and hand grip strength measurements in a subset of 24 patients using Spearman’s rank correlation coefficient (*ρ)*. We used one‐way analysis of covariance (ANCOVA) with post‐hoc Bonferroni adjustment for comparison of CK and Crn (dependent variables) between different patient subgroups considering the confounding factors. To match the assumptions of ANCOVA, we applied log transformation to the two dependent variables. We considered age, sex, weight, and height as covariates, because these variables differed significantly between the examined subgroups. For longitudinal analysis under nusinersen treatment, we performed Wilcoxon signed‐rank test to include all available data for the comparison between baseline and 18 ‐month follow‐up (representing forth maintenance dose). Datasets with missing values were excluded pairwise for cross‐sectional and longitudinal analyses using Wilcoxon signed‐rank test. To test for intraindividual longitudinal consistency, we used the Friedman test with post‐hoc Dunn‐Bonferroni adjustment, where only complete datasets with all time points from baseline to 18 months were included (CK: *n* = 58; Crn: *n* = 69). To explore cut‐off values and their accuracy of baseline CK and Crn for prediction of treatment response, receiver operating characteristic (ROC) curve and area under the curve (AUC) were calculated. Additionally, comparison of baseline CK and Crn levels between treatment responders and non‐responders was done using one‐way ANCOVA after applying log transformation considering the covariates given above. Concerning the comparison of CK changes within 18 months between subgroups, we applied reflect and square root transformation to the difference between CK at 18 months and baseline values to approach normality. Treatment responders were determined according to any increase (≥1) and non‐responders according to any decrease (≤ −1) on HFMSE score at 18‐month follow‐up compared to baseline. Patients with no change on the score were not included. Critical value was set as *p* < 0.05 two‐sided. An amount of Crn values were below the measuring range (e.g., for baseline Crn: *n* = 18). We did not exclude these values but took the lower limit of the measuring method as set.

## Results

**Figure 1:**
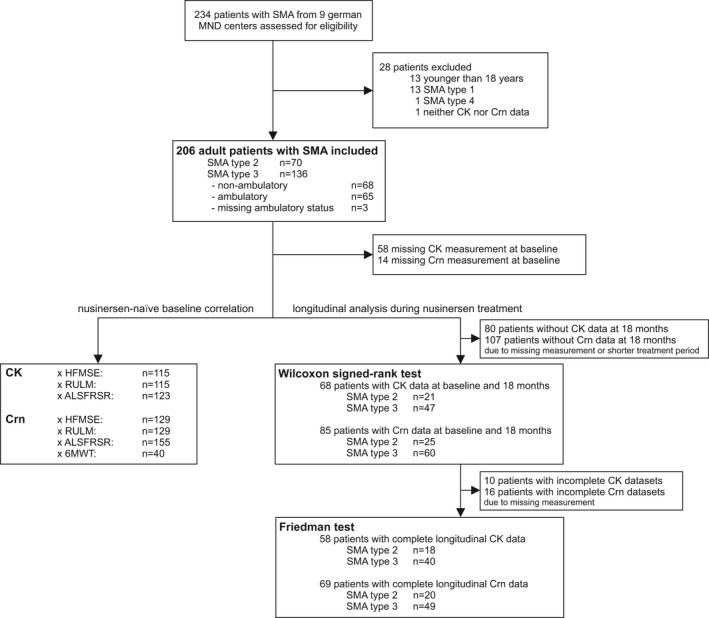
Study profile.

Two hundred and six adult patients with SMA type 2 (*n* = 70) and 3 (*n* = 136) were included in the analysis. Mean age was 36.2 years (± SD 12.5; range 18–71), and 41.3% were female. Details of study group characteristics and study profile are presented in Table 1 and Figure 1.

**Table 1:** Demographic features and baseline data.

Age [years]		
Mean ± SD (range)	36.2 ± 12.5 (18–71)	
Sex, *n* (%)		
Male	121 (58.7)	
Female	85 (41.3)	
SMA type, *n* (%)		
2	70 (34)	
3	136 (66)	
SMN2 copy number, *n* (%)		
2	15 (7.3)	
3	85 (41.3)	
4+	74 (35.9)	
Unknown	32 (15.5)	
Weight [kg]		
mean ± SD (range)	62.3 ± 20.8 (17–120)	
Height [cm]		
mean ± SD (range)	165.5 ± 13.1 (130–194)	
BMI [kg/m^2^]		
mean ± SD (range)	22.5 ± 6.2 (8.1–40.9)	
Mobility, *n* (%)		
Ambulatory	65 (31.5)	
Non‐ambulatory	138 (67.0)	
Unknown	3 (1.5)	
CK [U/L], *n* = 148		
mean ± SD (range)	259.66 ± 387.97 (13.0–2223.0)	
*n* (%)		
Normal	99 (66.9)	
Elevated^1^	16 (10.8)	
Strongly elevated^2^	33 (22.3)	
Crn [µmol/L], *n* = 192		
mean ± SD (range)	26.37 ± 17.73 (2.0–77.79)	
*n* (%)		
Normal	19 (9.9)	
Decreased	173 (90.1)	
HFMSE, *n* = 157		Median: 9
mean ± SD (range)	20.3 ± 21.3 (0–66)	
RULM, *n* = 156		Median: 20
mean ± SD (range)	20.8 ± 12.8 (0–37)	
ALSFRS‐R, *n* = 187		Median: 31
mean ± SD (range)	31.5 ± 9.3 (2–48)	
6MWT, *n* = 46		
mean (range)	388.35 (42–728)	

CK, serum creatine kinase activity; Crn, serum creatinine concentration; HFMSE, Hammersmith Functional Motor Scale Expanded (range 0–66); RULM, Revised Upper Limb Module (range 0–37); ALSFRS‐R, revised ALS‐Functional Rating Scale (range 0–48); 6MWT, Six Minute Walk Test.

^1^ Elevated: >189.6 U/L (male); >166.2 U/L (female).

^2^ Strongly elevated: >360 U/L.

### In nusinersen‐naïve adult patients with SMA, CK strongly correlates with motor function and disease severity status

**Figure 2:**
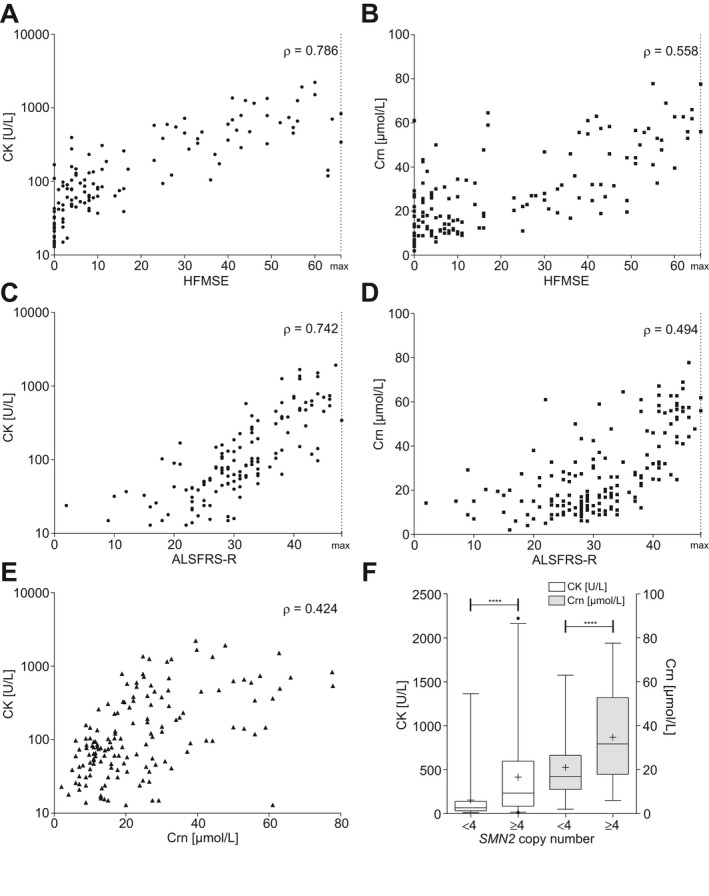
Partial and rank‐based correlation of CK/Crn to motor function and disease severity status in nusinersen‐naïve adult patients with SMA, corrected for sex, weight, and height. Correlation between CK (A and C)/Crn (B and D) and functional scores; Correlation between CK and Crn (E); Effect of *SMN2* copy number on CK/Crn (F), box and whisker plots show median (vertical line), mean (+), interquartile range (boxes), individual points illustrate values outside of 1.5 x interquartile range (whiskers) from the median. . ****p < 0.0001; HFMSE, Hammersmith Functional Motor Scale Expanded; ALSFRS‐R, revised ALS‐Functional Rating Scale; 6MWT, Six‐Minute Walk Test; CK, serum creatine kinase activity; Crn, serum creatinine concentration.

**Figure 3:**
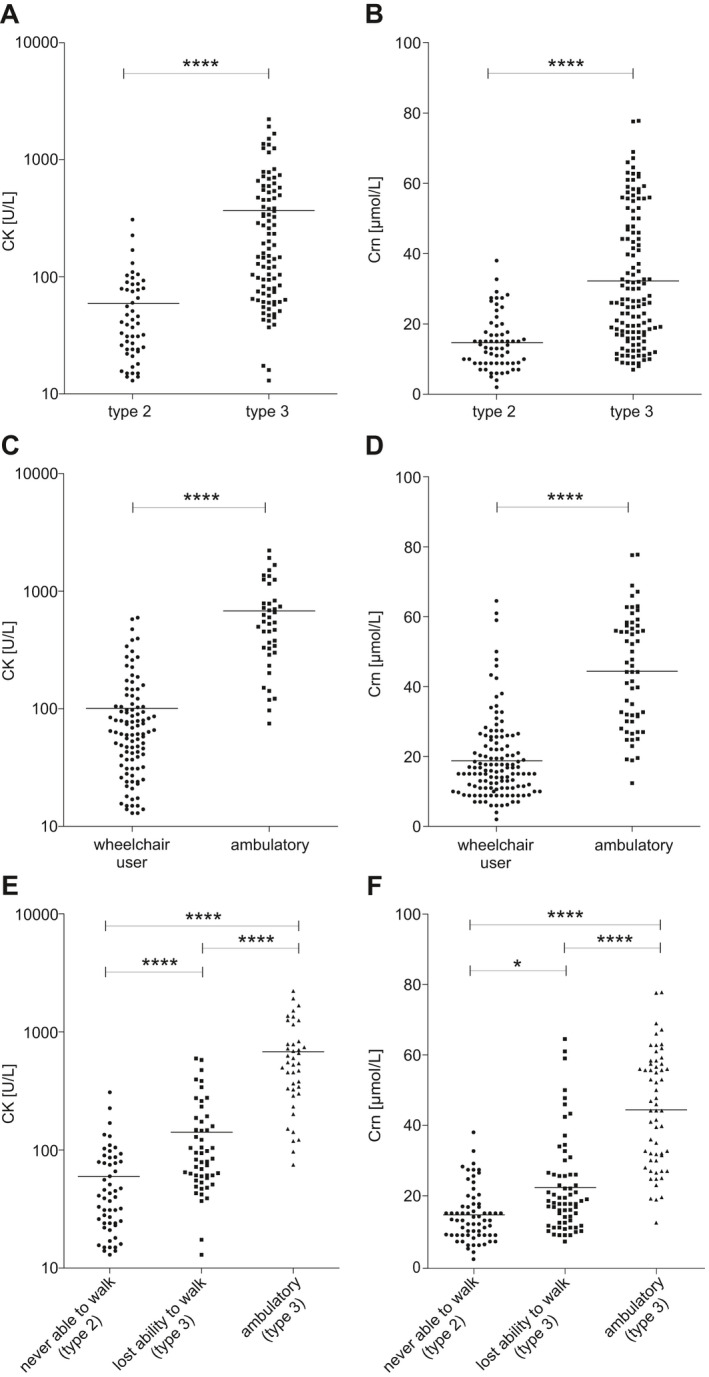
Comparison of CK/Crn between subgroups of adult patients with SMA regarding clinical subtypes (A and B) and mobility (C–F) examined by one‐way ANCOVA considering age, sex, weight, and height as covariates. Horizontal black line indicates the mean value; every symbol represents a single patient; *p < 0.05; **p < 0.01; ****p < 0.0001; CK, serum creatine kinase activity; Crn, serum creatinine concentration.

CK data from 148 patients were available at baseline before starting therapy with nusinersen. Referring to the German Society for Clinical Chemistry and Laboratory Medicine e.V. (normal range: female < 170 U/L; male < 190 U/L; standardized IFCC methods),^28^ CK initially was within the normal range in two thirds (66.9%) and elevated in one third of the patients (33.1%). CK was strongly correlated with HFMSE score (*ρ* = 0.786, *p* < 0.001), with RULM score (*ρ* = 0.736, *p* < 0.001), and with ALSFRS‐R score (*ρ* = 0.742, *p* < 0.001). CK and Crn showed moderate correlation (*ρ* = 0.424, *p* < 0.001) (for details see Table 2 and Fig. 2A, C, and E). No significant correlation to 6MWT assessment was observed (*p* = 0.426). CK was considerably higher in SMA type 3 (*p* < 0.0001) and ambulatory patients (*p* < 0.0001). Ambulatory patients with SMA type 3 revealed higher CK than patients with type 3 who already had lost the ability to walk (Fig. 3A, C, and E). Participants with four or more *SMN2* copies presented remarkably higher CK than those with less than four *SMN2* copies (*p* < 0.0001) as illustrated in Figure 2F.

**Table 2:** Partial and rank‐based correlations between CK/Crn and functional assessment corrected for sex, weight, and height.

		CK [U/L]	Crn [*µ*mol/L]
Crn [*µ*mol/L]	*ρ*=	0.424	
	*p* value	< 0.001	
	*n*=	140	
HFMSE	*ρ*=	0.786	0.558
	*p* value	< 0.001	< 0.001
	*n*=	115	129
RULM	*ρ*=	0.736	0.511
	*p* value	< 0.001	< 0.001
	*n*=	115	129
ALSFRS‐R	*ρ*=	0.742	0.494
	*p* value	< 0.001	< 0.001
	*n*=	123	155
6MWT	*ρ*=	0.154	0.575
	*p* value	n.s.	< 0.001
	*n*=	32	40

HFMSE, Hammersmith Functional Motor Scale Expanded (range 0–66); RULM, Revised Upper Limb Module (range 0–37); ALSFRS‐R, revised ALS‐Functional Rating Scale (range 0–48); 6MWT, Six‐Minute Walk Test; CK, serum creatine kinase activity; Crn, serum creatinine concentration.

### In nusinersen‐naïve adult patients with SMA, Crn moderately correlates with motor function and disease severity status

For the baseline analysis of Crn, datasets of 192 patients were available. Judging by the normal range described by Ceriotti et al.^29^ (female 44 – 80 *µ*mol/L; male 62 – 106 *µ*mol/L), Crn was mostly decreased (90.1%). Crn moderately correlated with HFMSE score (*ρ* = 0.558, *p* < 0.001), with RULM score (*ρ* = 0.511, *p* < 0.001), and with ALSFRS‐R score (*ρ* = 0.494, *p* < 0.001) (Table 2 and Fig. 2B and D). In contrast to CK, higher Crn did correlate with longer distances in the 6MWT assessment (*ρ* = 0.575, *p* < 0.001). Patients who had lost the ability to walk over time had remarkably lower Crn compared to patients who were still able to walk. At the same time, Crn was higher for patients who had lost the ability to walk than for patients who were never able to walk (Fig. 3B, D, and F). Participants with four or more *SMN2* copies presented considerably higher Crn than those with less than four *SMN2* copies (*p* < 0.0001) (Fig. 2F).

### In nusinersen‐naïve adult patients with SMA, CK and Crn correlate with MUNIX and hand grip strength

In a subgroup of the study cohort (patients of SMA care center Dresden; for demographic features and baseline data of the subset see Table S1), we studied associations of CK and Crn with denervation markers in treatment‐naïve patients. CK and Crn were strongly correlated with APB MUNIX (CK: *ρ* = 0.627, *p* < 0.01; Crn: *ρ* = 0.638, *p* < 0.01), APB MUSIX (CK: *ρ* = −0.614, *p* < 0.01; Crn: *ρ* = −0.697, *p* < 0.001), and hand grip strength (CK: *ρ* = 0.694, *p* < 0.0001; Crn: *ρ* = 0.586, *p* < 0.001) as well as moderately with APB CMAP (*ρ* = 0.580, *p* < 0.01) (Table S2 and Fig. S1). No correlation was found for Crn and APB CMAP.

### CK decreased in adult patients with SMA during nusinersen treatment, while motor function improved

**Figure 4:**
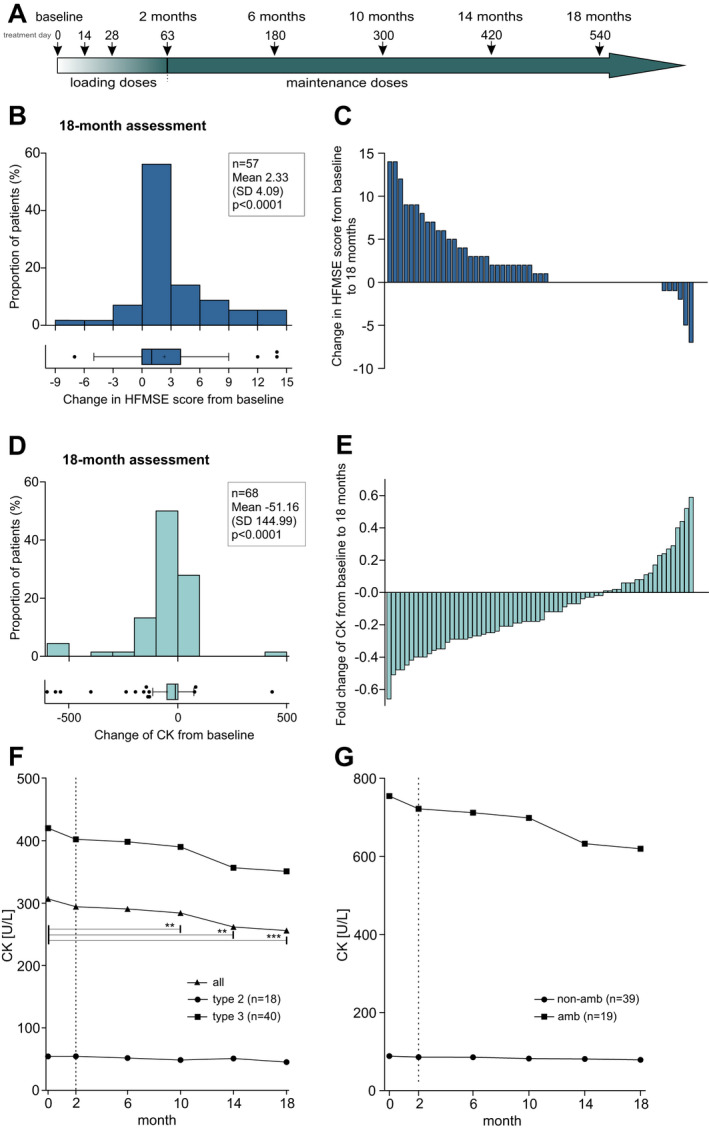
Longitudinal analysis of CK in adult patients with SMA during the observation period of the first 18 months under nusinersen treatment. (A) Dosing regimen proposed in prescribing information; (B) Mean changes in HFMSE score from baseline to 18 months, with each bar representing the proportion of patients related to the extent of score change. Box and whisker plots show median (vertical line), mean (+), and interquartile range (boxes), individual points illustrate values outside of 1.5× interquartile range (whiskers) from the median. (C) Change in HFMSE score with each bar representing a single patient. (D) Mean changes of CK from baseline to 18 months, with each bar representing the proportion of patients related to the extent of CK change. Box and whisker plots show median (vertical line), mean (+), and interquartile range (boxes), individual points illustrate values outside of 1.5× interquartile range (whiskers) from the median. (E) Fold change of CK referred to baseline value with each bar representing a single patient. (F) Longitudinal CK means (of complete longitudinal datasets) classified by clinical subtype (closed circle: SMA type 2, closed square: SMA type 3; closed triangle: all types); (G) Longitudinal CK means (of complete longitudinal datasets) classified by the ability to walk (closed circle: non‐ambulatory patients, closed square: ambulatory patients); dotted vertical line marks end of loading doses; CK, serum creatine kinase activity; HFMSE, Hammersmith Functional Motor Score Expanded; **: *p* < 0.01; ***: *p* < 0.001.

Comparable to our previously published study,^5^ motor function, assessed by the HFMSE score, improved from baseline to 18 months in the cohort of patients which could be included in the longitudinal analysis under nusinersen treatment (∆HFMSE: +2.33 ± 4.09, *p* < 0.0001; Table 3 and Fig. 4B and C). In this cohort, CK declined in 70.6% of the patients between the first and the last time points of the observation period (∆CK: −17.6%, *p* < 0.0001; Table 3 and Fig. 4D and E). The decline was consistent over time as shown by Friedman analysis (Fig. 4F and G and Table S3). The most relevant decrease of CK was found in SMA type 3 and ambulatory patients (Fig. 4F and G and Table 3).

**Table 3:** Changes in CK, Crn, and HFMSE during the observation period of 18 months (Wilcoxon signed‐rank test).

	*n*	Baseline mean ± SD	18‐month analysis mean ± SD	∆		*p* value
				abs.	%	
**CK [U/L]**	68	291.35 ± 408.89	240.19 ± 339.04	− 51.16	− 17.56	< 0.0001
SMA type 2	21	54.23 ± 32.10	45.39 ± 24.55	− 8.84	− 16.3	< 0.05
SMA type 3	47	397.30 ± 453.80	327.23 ± 379.96	− 70.07	− 17.64	< 0.001
non‐ambulatory	45	86.80 ± 75.62	77.82 ± 67.33	− 8.98	− 10.35	< 0.01
ambulatory	23	691.55 ± 494.69	557.87 ± 426.11	− 133.68	− 19.33	< 0.01
**Crn [*µ*mol/L]**	85	25.66 ± 1.90	26.88 ± 1.69	+ 1.22	+ 4.75	< 0.05
SMA type 2	25	15.52 ± 9.39	17.59 ± 8.84	+ 2.07	+ 13.34	n.s.
SMA type 3	60	29.89 ± 18.39	30.75 ± 16.14	+ 0.86	+ 2.88	n.s.
non‐ambulatory	55	16.96 ± 9.17	19.59 ± 8.65	+ 2.63	+ 15.51	< 0.01
ambulatory	30	41.63 ± 17.96	40.26 ± 16.57	− 1.37	− 3.29	n.s.
**HFMSE (out of 66)**	57	23.4 ± 21.0	25.7 ± 22.7	+ 2.3	+ 9.8	< 0.0001

CK, serum creatine kinase activity; Crn, serum creatinine concentration; HFMSE, Hammersmith Functional Motor Scale Expanded (range 0–66).

### Crn slightly increased in adult patients with SMA during nusinersen treatment

Crn marginally increased between the first and the last time point in 60% of the patients (Table 3 and Fig. S2A and B). Non‐ambulatory patients presented a notable increase comparing to the baseline level (+15.51%, *p* < 0.01) (Table 3; Fig. S2D; Table S3).

### Potential and accuracy of CK and Crn as predictive biomarkers

After adjustment for weight, height, and age, mean values of baseline CK and Crn in the group of treatment responders (CK (*n* = 25): 500.90 U/L ± 526.40; Crn (*n* = 29): 32.01 *µ*mol/L ± 17.09) were remarkably higher compared to the group of non‐responders (CK (*n* = 6): 96.53 U/L ± 48.88; Crn (*n* = 6): 14.67 *µ*mol/L ± 6.53; *p* < 0.05, respectively, Fig. S3C and D). ROC curve analysis revealed a cut‐off point for CK ≥ 99.5 U/L predicting treatment response, defined as gain of motor function assessed by an increase on HFMSE score, providing a sensitivity of 0.720 and a specificity of 0.833, and a cut‐off point for Crn ≥ 19.3 *µ*mol/L with a sensitivity of 0.759 and a specificity of 0.833. The AUC was 0.773 (*p* < 0.01) for CK and 0.833 (*p* < 0.05) for Crn (Fig. S3A). As shown in Figure S3B and E, patients with an increase on the HFMSE score indicating treatment response within 18 months present a more pronounced decline of CK (∆CK: −20.5%, *p* < 0.01; Fig. S3E) than patients classified as non‐responders (∆CK: −11.1%, n.s.; Fig. S3E). For Crn, non‐responders revealed a distinct increase of Crn (∆Crn: +25.0%, *p* < 0.05; Fig. S3F), while patients with improved HFMSE score show a negligible increase of Crn (∆Crn: +5.3%, n.s.; Fig. S3F). However, the small sample size (resulting of missing biomarker and motor score data) compromises the longitudinal dataset, and further prospective studies are needed to analyze these longitudinal findings in depth.

## Discussion

Various novel gene/RNA‐modifying and gene replacement therapies are currently revolutionizing the therapeutic landscape in SMA. Decisions about treatment initiation, change of treatment, or the discontinuation of treatment will be challenging, particularly in patients with long disease duration. Although the motor scores applied in this study (HFMSE and RULM) are validated for the examination of the motor function of patients with SMA,^30^, ^31^ they have some limitations when used in clinical routine. Most relevant, patients with severe impairment often cannot execute the tasks demanded by the HFMSE, which results in a substantial floor effect (15% in our cohort with score of 0) and, on the other hand, mildly affected patients are able to perform every item of the RULM and reach full scores, which consequently generates a ceiling effect (24% in our cohort with maximum score). Furthermore, these scores are time‐consuming, require well‐trained raters, and outside of controlled study conditions, motor scoring might be vulnerable to inter‐rater and even intra‐rater variability in daily clinical practice. Therefore, objective prognostic biomarkers for disease severity assessment and predictive biomarkers for therapy evaluation are needed in addition.

Besides electrophysiological,^32^ imaging,^33^, ^34^ or other “hardware‐based” markers of, for example, muscle strength or pulmonary function, biomarkers derived from serum, CSF, or other liquid bioanalytes would be most helpful. The number of *SMN2* copies is already applied for a reliable but rough division of disease severity, as it is known to be the main modifier of the clinical course of the disease. However, the *SMN2* copy number represents a static factor, useful for a prognosis at baseline but not applicable to assess disease dynamics. Furthermore, there are relevant limitations, as patients with three *SMN2* copies show a wide variability of clinical appearance and reveal phenotypic overlap with patients with four *SMN2* copies.^35^ Circulating SMN protein might be more suitable and was shown to be associated with disease severity and the degree of denervation; nevertheless, blood SMN protein level did not change during therapy with nusinersen or gene therapy.^13^, ^36^, ^37^ Neurofilament levels are very promising objective circulating biomarkers^38^, ^39^; however, serum and cerebrospinal fluid (CSF) neurofilament levels in adult patients with SMA are not valuable based on recent pilot studies,^40^, ^41^, ^42^ although final conclusions can only be made based on larger studies with longer observation periods. Levels of neuron‐specific enolase and phosphorylated Tau‐protein, both measured in the CSF, were shown to decrease noticeably under nusinersen treatment indicating an alleviation of axonal injury over time^7^; however, their potential as biomarkers has not yet been further investigated. Interestingly, a recent mass spectrometry‐based CSF proteomic analysis demonstrated in a small cohort of adult patients with later‐onset SMA that clustering of patient groups according to distinct molecular signaling networks in correlation with clinical outcome parameters is feasible. However, this study did not consistently identify a single CSF protein to be differentially expressed in response to nusinersen treatment.^43^ Investigations of markers of muscle destruction and muscle metabolism in SMA are sparse, especially in adult patients and, most importantly, not systematically done within one population of genetically defined patients also respecting *SMN2* copy numbers.

In this multicenter study, we comprehensively investigated CK and Crn in relation to different clinical features cross‐sectionally and additionally longitudinally under nusinersen treatment in adult patients with SMA. One third of patients with SMA in our study cohort showed elevated CK, which is in line with previous research.^23^ Since the majority of creatine kinase is located in the skeletal muscle, the elevation of CK is most likely due to an increased enzyme leakage resulting from muscle damage or myolysis caused by muscle atrophy and/or secondary myopathic changes due to the increased muscle work burden of the remaining vulnerable muscles.^44^ However, the standard reference range of healthy people is not suitable for most patients with SMA because of the strongly reduced muscle mass resulting in reduced total amount of creatine kinase compared to healthy controls. In consequence, the number of patients with increased CK presumably was estimated too low. Almost all patients in our study cohort presented decreased Crn, similar to the results of a recent study.^22^ This is most likely caused by the reduced muscle mass due to the remarkable muscle atrophy and/or impaired muscle energy metabolism due to the leakage of different cytosolic components.^18^, ^44^

Better motor function, stronger hand grip strength, less denervation status, and better feasibility of everyday life situations were associated with higher CK and Crn. Participants with four or more *SMN2* copies had higher CK and Crn than those with less than four *SMN2* copies. This finding has not previously been described for CK, but has already been reported for Crn.^22^ In line with earlier investigations,^22^, ^23^ our data show an extensive difference of CK and Crn between clinical subtypes, underpinning their value as suitable biomarkers to assess disease severity and to distinguish between the established subtypes of SMA. Moreover, we identified the most pronounced differences of CK and Crn between groups of patients with SMA with regard to their mobility. Patients who were still able to walk had considerably higher CK and Crn than patients using a wheelchair, even within the group of patients with SMA type 3. For Crn, this has already been reported by Alves et al. particularly with regard to children and adolescents,^22^ but with limited information relating to adult patients. In regard to CK, this finding is inconsistent with a previous study.^23^ Interestingly, there were noticeable differences of CK and Crn within the group of wheelchair users between patients who previously were able to walk and lost this ability (type 3) and those who were never able to walk (type 2 by definition). This finding indicates a relation between CK/Crn and disease severity besides the effect caused by the patients’ individual level of activity. Furthermore, CK and Crn showed a remarkable correlation, demonstrating that more preserved muscle mass implies a higher amount of intracellular creatine kinase, leading to more creatinine metabolites.

Similar to previous research in SMA children,^22^ lower Crn was associated with worsened electrophysiological measurement values in SMA adults. Our data revealed a strong positive correlation between CK/Crn and the number of motor units and a clear negative correlation with the size of motor units of the APB, which characterizes denervation and reinnervation following axonal damage as already observed in SMA, substantiating the value of CK and Crn for determining disease severity. We studied the APB, because it was shown to be relatively preserved in adult patients with SMA allowing more reliable information about the neural damage of variously affected individuals.^32^

Longitudinal analyses, examining intraindividual changes within our observation period of the first 18 months under nusinersen treatment, revealed a consistent decrease of CK in combination with a slightly increased course of Crn, while motor function improved concurrently. With regard to Crn, the natural history disease course (without disease‐modifying treatment)^22^ leads to Crn decrease over time. Additionally, we were able to set cut‐off values for baseline CK and Crn to predict nusinersen treatment response (CK cutoff: 99.5 U/L ‐ sensitivity 72%/specificity 83%; Crn cutoff: 19.3 *µ*mol/L – sensitivity 76%/specificity 83%). Nusinersen treatment may lead in consequence of stabilized motor units to improved muscle integrity, including reduced leakage of components of intracellular energy metabolism of muscle cells (e.g., creatine kinase). This might lead to a higher turnover of phosphocreatine to creatine which is catalyzed by creatine kinase. Creatinine originates in a spontaneous, non‐enzymatic process during the degradation of creatine and phosphocreatine and might—in consequence of a higher intracellular creatine kinase activity— accumulate in serum prior to renal elimination.

To the best of our knowledge, this is the first study which shows nusinersen‐depending effects on disease‐relevant fluid biomarkers in adult patients with SMA.

CK is influenced by muscle mass and muscle damage. Both more muscle mass and increased vulnerability of the muscle lead to higher release of creatine kinase from myocytes into serum, which accounts for the higher CK levels presented by SMA patients with better motor function in the baseline analysis (treatment naïve). Fitting to this, SMA walkers show CK levels above the upper limit of the reference range, which illustrates the vulnerability of the SMA muscle. Therefore, both factors have to be considered in the interpretation of CK levels and dynamics in our SMA cohort. Of note, the reference range is determined for healthy individuals with normal muscle mass, and SMA walkers already suffer from strong muscle wasting. During treatment course, we observed a significant decrease of CK toward “normal” values, which we thus interpret as a stabilization of the vulnerable muscle rather than a decrease in muscle mass. Additionally, by monitoring creatinine (strong marker of muscle mass), patients do not seem to lose muscle mass. The interpretation of the CK dynamic in severe affected SMA patients is more difficult due to the strongly reduced muscle mass with per se lower CK levels.

While the reported CK/Crn dynamics suggest therapy response in our nusinersen‐treated cohort, Tiziano et al. reported an increase of CK associated with increasing *SMN2*‐full length transcripts in SMA patients treated with salbutamol in a clinical trial.^45^ Here, CK values were evaluated as part of safety assessment, because salbutamol and other beta2 agonists are known to increase CK levels^46^ regardless of the underlying disease (also patients without neuromuscular diseases^47^, ^48^). This is of interest, as it shows that CK is influenced by different factors, which have to be properly controlled in further prospective studies.

Our study has some limitations. Due to the nature of a retrospective design, there have been numerous missing data (as listed in Table 1). Some Crn values were below the detection range of the Jaffe method, hence producing a floor effect, especially within the dataset of patients with SMA type 2. This was not the case when using a comparable enzymatic method,^26^ but that was not available in all centers. We did not exclude these values but took the lower limit of the detection method as set. Renal function was not systematically monitored with Crn‐independent methods; however, in a small subset of patients, no changes in cystatin C‐calculated glomerular filtration rate were observed under nusinersen treatment (data not shown). In addition, we did not acquire body weight or lean body mass longitudinally. Future studies should strive for a prospective design considering all known influencing factors, particularly with regard to lean body mass.

In conclusion, we comprehensively studied the potential of CK and Crn as disease and treatment response biomarkers in adult, long‐term diseased patients with SMA. Our data show that both CK and Crn reflect the severity of SMA and might serve as useful and easily accessible biomarkers to predict treatment response and to monitor the disease course and could therefore be implemented effortless in the laboratory routine of the monitoring of patients with SMA. During nusinersen treatment, CK decreased and Crn slightly increased, while motor function ameliorated coincidently in adult patients with SMA type 2 and type 3 based on motor score improvements.^5^, ^6^, ^7^, ^8^, ^10^, ^49^ To what degree CK levels depend on muscle mass or muscle damage in SMA and whether a therapy decision could be made based on the dynamic of CK or/and Crn should be evaluated in further prospective studies. We suggest monitoring CK and Crn as additional laboratory assessments.

## Conflict of Interest

M. Freigang reports non‐financial support from Biogen outside the submitted work. C. D. Wurster has received honoraria from Biogen as an advisory board member and for lectures and as a consultant and advisory board member from Hoffmann‐La Roche. She also received travel expenses from Biogen. T. Hagenacker received research support from Biogen, AveXis, and Hoffmann‐La Roche and Sanofi‐Genzyme; consultant fees, speaker honoraria, and ad board compensation from Biogen, Novartis, Hoffmann‐La Roche, PTC, Akcea, and Sanofi‐Genzyme. M. Weiler received advisory board and/or speaker honoraria and financial support for conference attendances from Akcea Therapeutics, Alnylam Pharmaceuticals, Biogen, and Pfizer, and advisory board honoraria from Hoffmann‐La Roche. C. Kamm and B. Stolte have received personal fees from Biogen outside of the submitted work. O. Schreiber‐Katz received honoraria as a speaker/consultant and/or funding for travel expenses from the German Neuromuscular Society (Deutsche Gesellschaft fuer Muskelkranke, DGM e.V.), Novartis, Biogen GmbH, the Jain Foundation, and Biermann Verlag GmbH; and research support from the DGM e.V. A. Osmanovic has received honoraria as speaker/consultant from Biogen. S. Petri has received grants from the German Neuromuscular Society, the Federal Ministry of Education and Research, the German Israeli Foundation for Scientific Research and Development, and the EU Joint Programme for Neurodegenerative Disease Research; and other support from Cytokinetics, Desitin Pharma, Biogen, Novartis, and Teva outside of the submitted work. A. Kowski reports no disclosures. T. Meyer received advisory board honoraria from Biogen, Mitsubishi Tanabe, Desitin, Tilray, and Cytokinetics. He is co‐founder of the internet platform Ambulanzpartner and hold shares in Ambulanzpartner Soziotechnologie APST GmbH. J. C. Koch has received payment for consultation and advisory board participation from Biogen, Hoffmann‐La Roche, and AveXis. I. Cordts has received grants from Biogen outside of the submitted work. M. Deschauer received fees for advisory board participations from Biogen and Hoffmann‐La Roche. P. Lingor has received support for symposium organization from Biogen; as well as speaker honoraria from Desitin, BIAL, and AbbVie, and fees for advisory board participation from Novartis outside of the submitted work. E. Aust reports no disclosures. D. Petzold reports non‐financial support from Biogen outside the submitted work. A. C. Ludolph has received personal fees from AB Science, Biogen, Cytokinetics, GlaxoSmithKline, Orion Pharma, Novartis, Tau Rx Therapeutics, Teva, Mitsubishi, and Hoffmann‐La Roche outside of the submitted work. B. Falkenburger reports no disclosures. A. Hermann has received personal fees and non‐financial support from Biogen and Desitin during the conduct of the study; and grants from the Helmholtz Foundation, the Federal Ministry of Education and Research, Innovationsausschuss des G‐BA, the German Neuromuscular Society, and the Schilling‐Stiftung outside of the submitted work. R. Günther has received honoraria from Biogen as an advisory board member and for lectures and as a consultant and advisory board member from Hoffmann‐La Roche. He also received travel expenses and research support from Biogen.

## Authors Contributions

**Table:** 

Name	Location	Contribution
Maren Freigang	Department of Neurology, Universitätsklinikum Carl Gustav Carus, Technische Universität Dresden, Dresden, Germany	Acquisition, analysis and interpretation of data; writing—original draft preparation, revising the manuscript
Claudia D Wurster, MD	Department of Neurology, Ulm University, Ulm, Germany	acquisition of data, revising the manuscript for intellectual content
Tim Hagenacker, MD	Department of Neurology, University Hospital Essen, Essen, Germany	acquisition of data, revising the manuscript for intellectual content
Benjamin Stolte, MD	Department of Neurology, University Hospital Essen, Essen, Germany	acquisition of data, revising the manuscript for intellectual content
Markus Weiler, MD	Department of Neurology, Heidelberg University Hospital, Heidelberg, Germany	acquisition of data, revising the manuscript for intellectual content
Christoph Kamm, MD	Department of Neurology, University of Rostock, Rostock, Germany	acquisition of data, revising the manuscript for intellectual content
Olivia Schreiber‐Katz, MD	Department of Neurology, Hannover Medical School, Hannover, Germany	acquisition of data, revising the manuscript for intellectual content
Alma Osmanovic, MD	Department of Neurology, Hannover Medical School, Hannover, Germany	acquisition of data, revising the manuscript for intellectual content
Susanne Petri, MD	Department of Neurology, Hannover Medical School, Hannover, Germany	acquisition of data, revising the manuscript for intellectual content
Alexander Kowski, MD	Department of Neurology, Center for ALS and other Motor Neuron Disorders, Charité Universitätsmedizin Berlin, Berlin, Germany	acquisition of data, revising the manuscript for intellectual content
Thomas Meyer, MD	Department of Neurology, Center for ALS and other Motor Neuron Disorders, Charité Universitätsmedizin Berlin, Berlin, Germany	acquisition of data, revising the manuscript for intellectual content
Jan C Koch, MD	Department of Neurology, University Medicine Göttingen, Göttingen, Germany	acquisition of data, revising the manuscript for intellectual content
Isabell Cordts, MD	Department of Neurology, Klinikum Rechts der Isar, Technical University of Munich, Munich, Germany	acquisition of data, revising the manuscript for intellectual content
Marcus Deschauer, MD	Department of Neurology, Klinikum Rechts der Isar, Technical University of Munich, Munich, Germany	acquisition of data, revising the manuscript for intellectual content
Paul Lingor, MD	Department of Neurology, Klinikum Rechts der Isar, Technical University of Munich, Munich, Germany	acquisition of data, revising the manuscript for intellectual content
Elisa Aust, M. Sc.	Department of Neurology, Universitätsklinikum Carl Gustav Carus, Technische Universität Dresden, Dresden, Germany	analysis of data, revising the manuscript for intellectual content
Daniel Petzold	Department of Neurology, Universitätsklinikum Carl Gustav Carus, Technische Universität Dresden, Dresden, Germany	acquisition of data, revising the manuscript for intellectual content
Albert C Ludolph, MD	Department of Neurology, Ulm University, Ulm, Germany; German Center for Neurodegenerative Diseases (DZNE) Ulm, Ulm, Germany	acquisition of data, revising the manuscript for intellectual content
Björn Falkenburger, MD	Department of Neurology, Universitätsklinikum Carl Gustav Carus, Technische Universität Dresden, Dresden, Germany; German Center for Neurodegenerative Diseases (DZNE) Dresden, Dresden, Germany	acquisition of data, revising the manuscript for intellectual content
Andreas Hermann, MD, PhD	Translational Neurodegeneration Section „Albrecht‐Kossel“, Department of Neurology, University Medical Center Rostock, University of Rostock, Rostock, Germany; German Center for Neurodegenerative Diseases (DZNE) Rostock/Greifswald, Rostock, Germany	Conception and design of the work; acquisition and analysis of data, revising the manuscript for intellectual content
René Günther, MD	Department of Neurology, Universitätsklinikum Carl Gustav Carus, Technische Universität Dresden, Dresden, Germany; German Center for Neurodegenerative Diseases (DZNE) Dresden, Dresden, Germany	Conception and design of the work; writing—original draft preparation; revising the manuscript; supervision; project administration

## Supporting information


**Figure S1**. Correlations of CK/Crn to (A, B) hand grip strength [kg] and (C‐H) electrophysiological values in nusinersen‐naïve adult patients with SMA (*n* = 23).

**Figure S2**. Longitudinal analysis of Crn during 18 months of nusinersen treatment (A‐D). Reverse dynamics of CK ad Crn during our observational period.

**Figure S3.** Applicability of CK / Crn to predict treatment response prior to treatment initiation (A, C, D). Change of CK / Crn within 18 months of nusinersen treatment with regard to display treatment response (B, E, F).

**Table S1.** Demographic features of a subset of nusinersen‐naïve adult SMA patients (*n* = 24) with hand grip strength and electrophysiological measures.
**Table S2.** Correlations between CK/Crn and hand grip strength and electrophysiological measurement in nusinersen‐naïve adult patients with SMA.
**Table S3.** Changes in CK, Crn and HFMSE during the observation period of 18 months after listwise exclusion of data using Friedman test with post‐hoc Dunn‐Bonferroni adjustment.
