# Supplementary figures and images for: Serum creatine kinase and creatinine in adult spinal muscular atrophy under nusinersen treatment
Source: Ann Clin Transl Neurol. 2021 Mar 31;8(5):1049–63. doi: 10.1002/acn3.51340 (PMC8108420; doi:10.1002/acn3.51340)

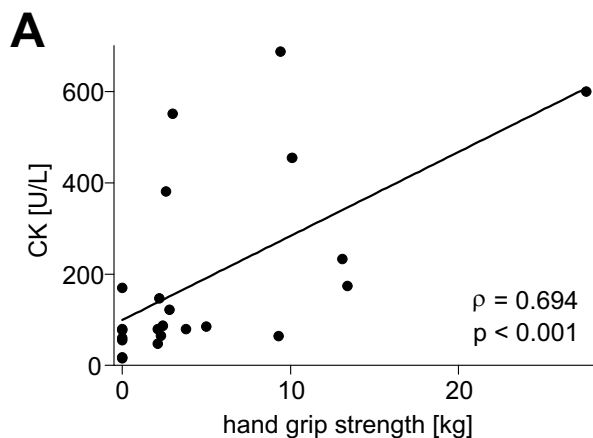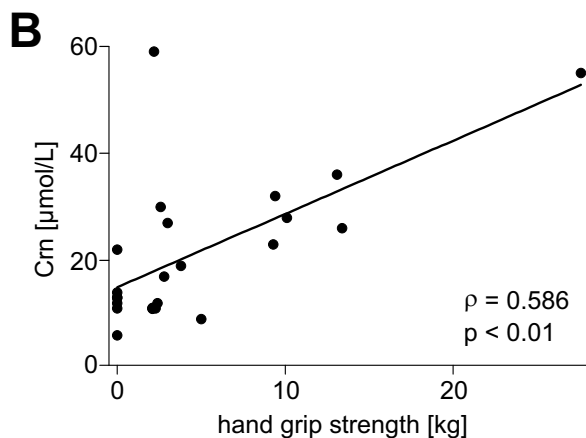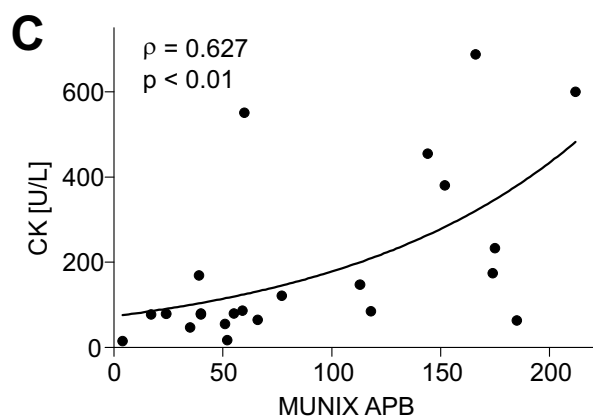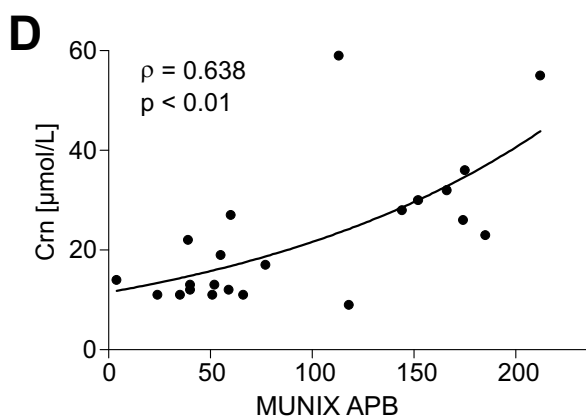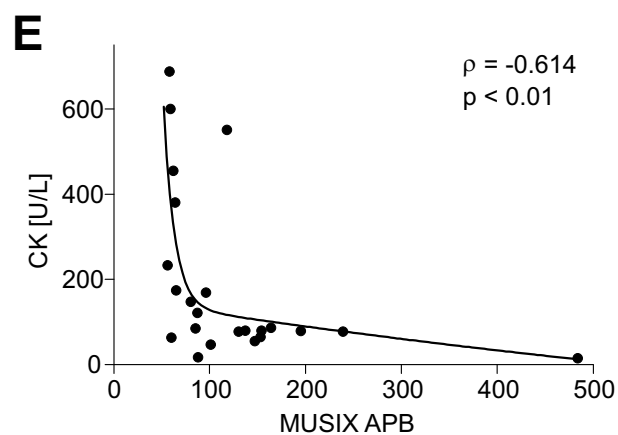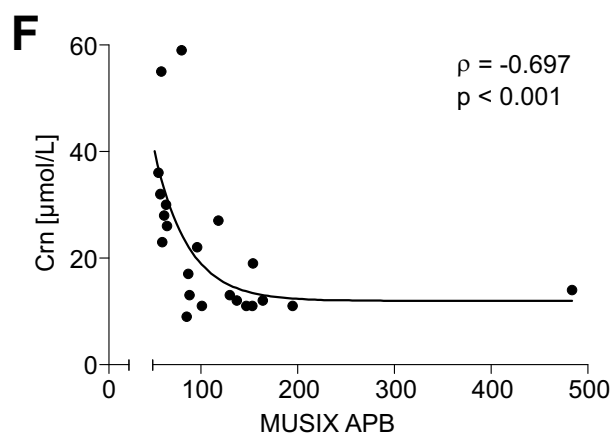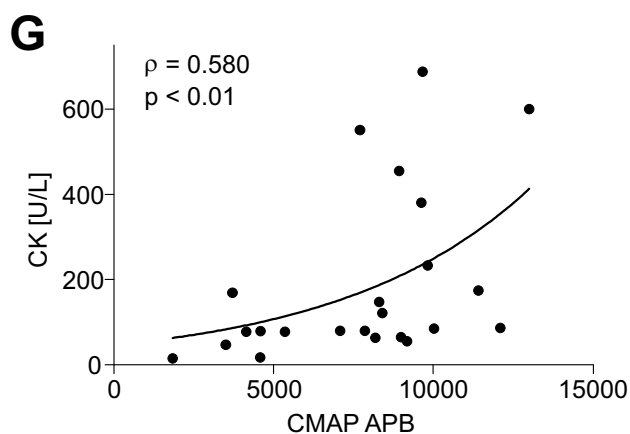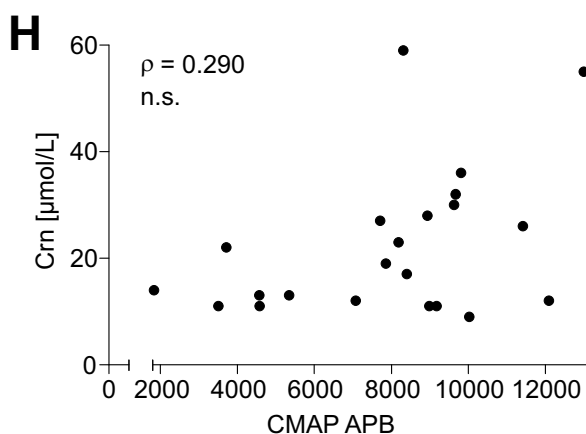

Supplement: Supplementary file 1 — Figure S1. Correlations of CK/Crn to (A, B) hand grip strength [kg] and (C‐H) electrophysiological values in nusinersen‐naïve adult patients with SMA (n = 23). [file ACN3-8-1049-s002.pdf]

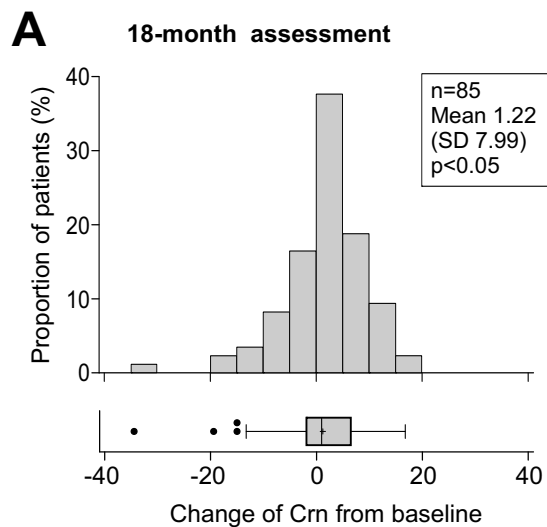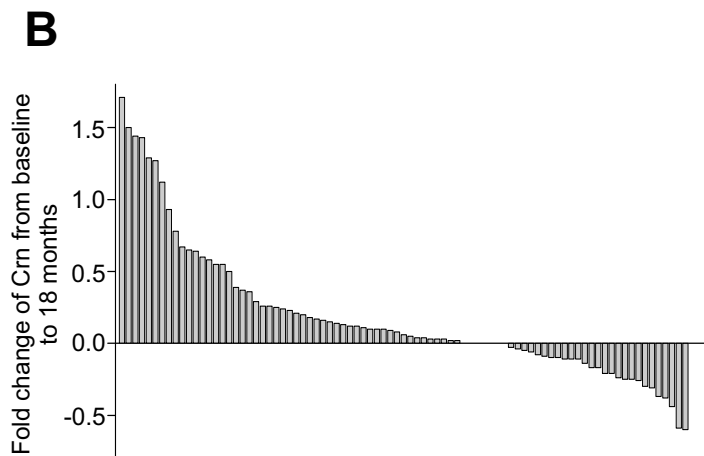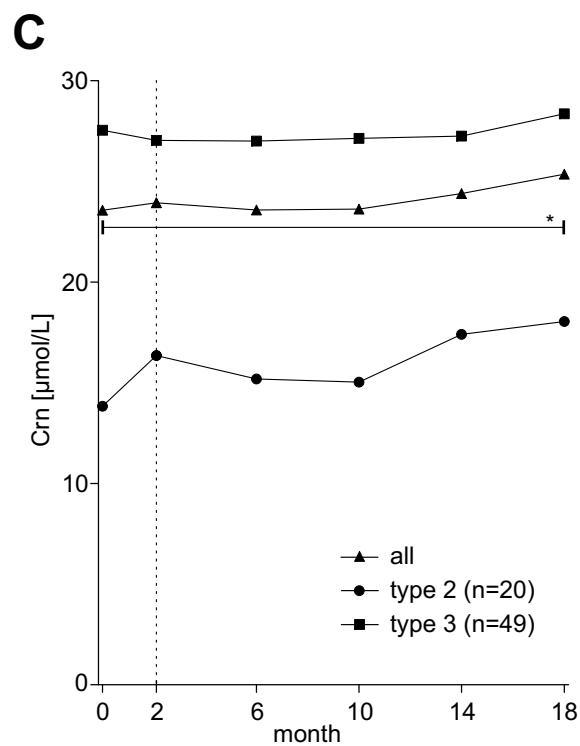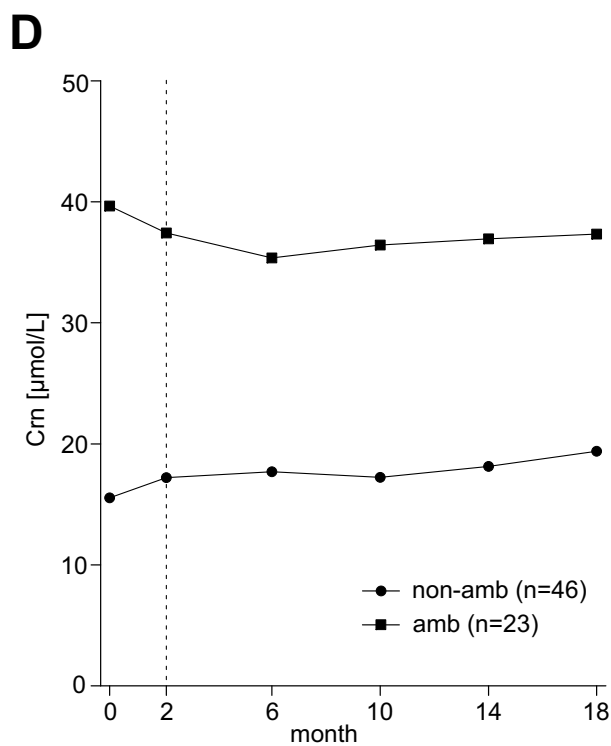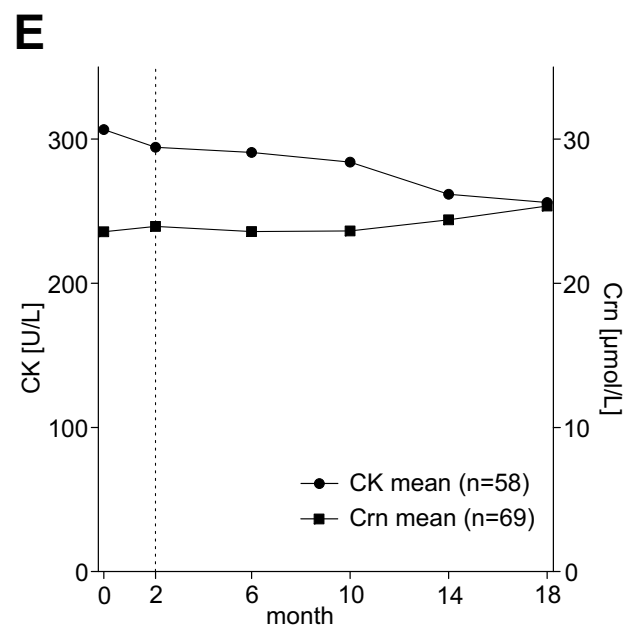

Supplement: Supplementary file 2 — Figure S2. Longitudinal analysis of Crn during 18 months of nusinersen treatment (A‐D). Reverse dynamics of CK ad Crn during our observational period. [file ACN3-8-1049-s004.pdf]

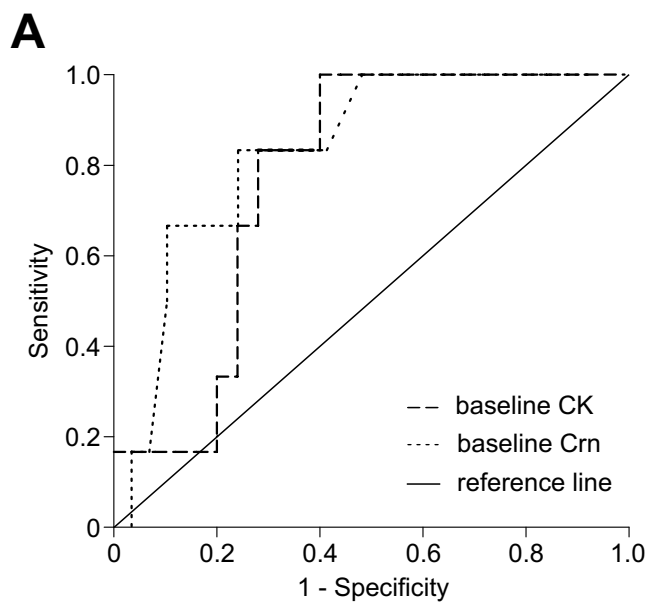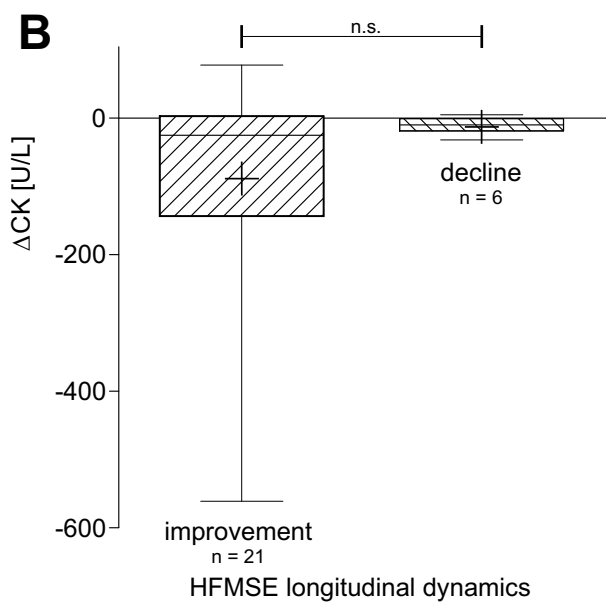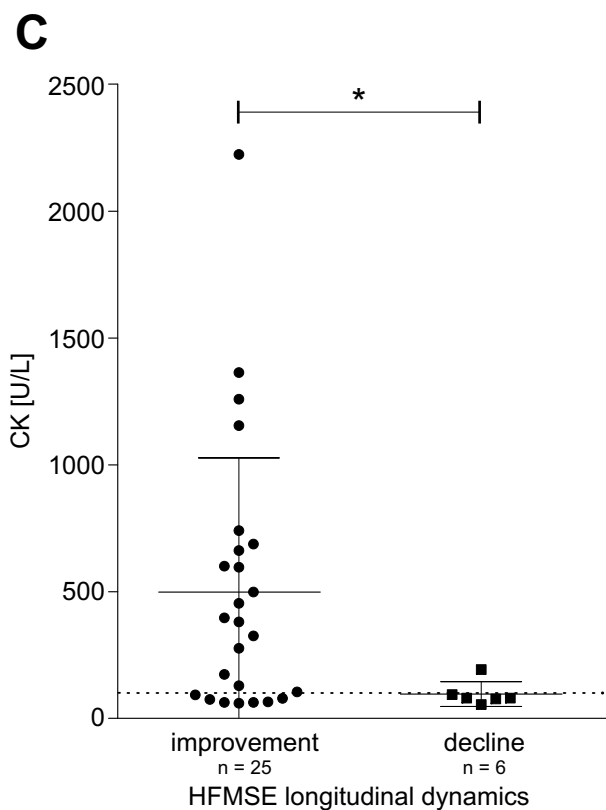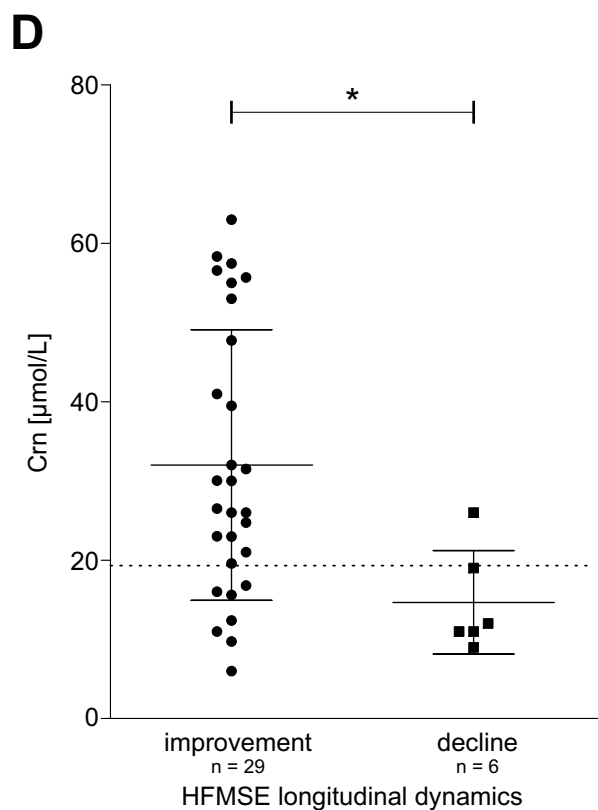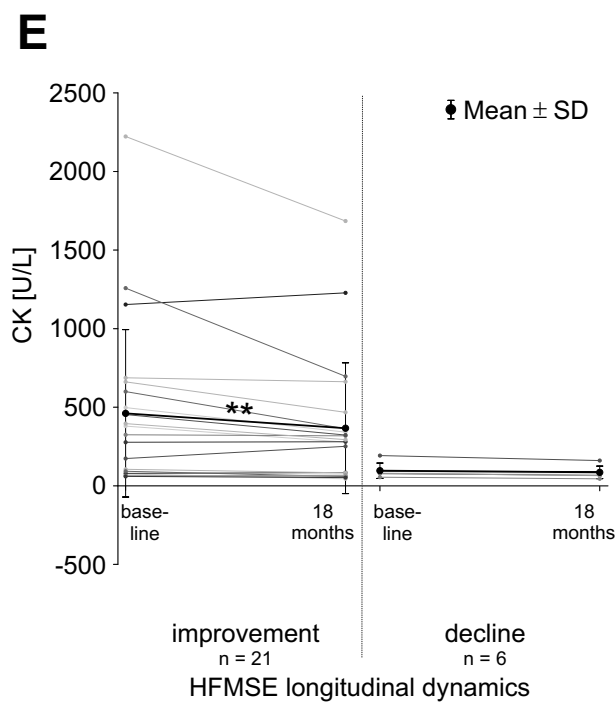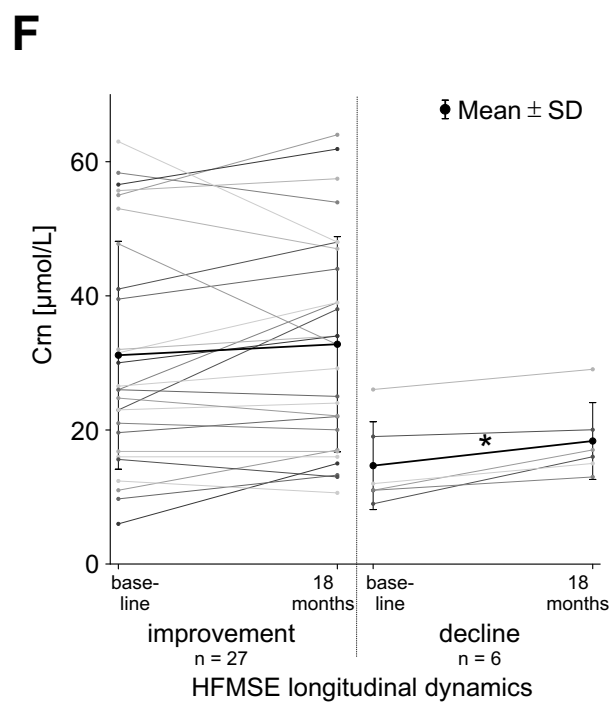

Supplement: Supplementary file 3 — Figure S3. Applicability of CK / Crn to predict treatment response prior to treatment initiation (A, C, D). Change of CK / Crn within 18 months of nusinersen treatment with regard to display treatment response (B, E, F). [file ACN3-8-1049-s003.pdf]
