# Supplementary material for: Serum creatine kinase and creatinine in adult spinal muscular atrophy under nusinersen treatment
Source: Ann Clin Transl Neurol. 2021 Mar 31;8(5):1049–63. doi: 10.1002/acn3.51340 (PMC8108420; doi:10.1002/acn3.51340)
Supplement: Supplementary file 4 — Table S1. Demographic features of a subset of nusinersen‐naïve adult SMA patients (n = 24) with hand grip strength and electrophysiological measures. Table S2. Correlations between CK/Crn and hand grip strength and electrophysiological measurement in nusinersen‐naïve adult patients with SMA. Table S3. Changes in CK, Crn and HFMSE during the observation period of 18 months after listwise exclusion of data using Friedman test with post‐hoc Dunn‐Bonferroni adjustment. [file ACN3-8-1049-s001.docx]

**supplemental Figure legends**

**Suppl. Figure S1**

Correlations of CK/Crn to (A, B) hand grip strength [kg] and (C-H) electrophysiological values in nusinersen-naïve adult patients with SMA (n=23)

CK = serum creatine kinase activity; Crn = serum creatinine concentration

MUNIX = motor unit number index; MUSIX = motor unit size index; CMAP = compound muscle action potential; APB = abductor pollicis brevis muscle;

ρ = Spearman’s rank correlation coefficient

**Suppl. Figure S2**

(A) Longitudinal analysis of Crn in adult patients with SMA during the observation period of the first 18 months under nusinersen treatment. Mean changes of Crn from baseline to 18 months, with each bar representing the proportion of patients related to the extent of Crn change. Box and whisker plots show median (vertical line), mean (+), interquartile range (boxes), individual points illustrate values outside of 1.5 x interquartile range (whiskers) from the median. (B) Fold change of Crn referred to baseline value with each bar represents a single patient.

(C) Longitudinal Crn means (of complete data sets) classified by clinical subtype (closed circle: SMA type 2, closed square: SMA type 3; closed triangle: all types); (D) Longitudinal Crn means (of complete data sets) classified by the ability to walk (closed circle: non-ambulatory patients, closed square: ambulatory patients); (E) Longitudinal CK (closed circle) and Crn (closed square) means of complete data sets; dotted vertical line marks end of loading doses;

CK = serum creatine kinase activity; Crn = serum creatinine concentration; *: p<0.05

**Suppl. Figure S3**

(A) Receiver operating characteristic (ROC) curve analysis to assess the accuracy of baseline CK and Crn as predictors of nusinersen treatment response defined by gain or loss on HFMSE score. (B) CK change between baseline and T8 comparing treatment responders (ascending striped box) to non-responders (descending striped box) calculated by one-way ANCOVA corrected for age, weight and height. Box and whisker plots show median (central line), mean (+), interquartile range (boxes), whiskers extend minimum and maximum value. (C) Comparison of baseline CK between treatment-responders (closed circle) and non-responders (closed square) calculated by one-way ANCOVA corrected for age, weight and height. Each icon represents a single patient. Central line marks mean value; whiskers present standard deviation. Dotted line demonstrates cutoff value determined by ROC curve analysis. (D) Comparison of baseline Crn between treatment-responders (closed circle) and non-responders (closed square) calculated by one-way ANCOVA corrected for age, weight and height. Each icon represents a single patient. Central line marks mean value; whiskers present standard deviation. Dotted line demonstrates cutoff value determined by ROC curve analysis. (E) Paired longitudinal dynamics of CK during 18 months of nusinersen treatment plotted separately regarding treatment-response. Each grey line represents a single patient. Black closed circles show mean values, whiskers illustrate standard deviation. Asterisk states significance related to the difference between baseline and 18-month follow-up calculated by Wilcoxon signed-rank test. (F) Paired longitudinal dynamics of Crn during 18 months of nusinersen treatment plotted separately regarding treatment-response. Each grey line represents a single patient. Black closed circles show mean values, whiskers illustrate standard deviation. Asterisk states significance related to the difference between baseline and 18-month follow-up calculated by Wilcoxon signed-rank test. CK = serum creatine kinase activity; Crn = serum creatinine concentration; HFMSE = Hammersmith Functional Motor Score Expanded; *: p<0.05; **: p<0.01

**SUPPLEMENTAL TABELS**

**Suppl. Table S1** Demographic features of a subset of nusinersen- naïve adult SMA patients (n=24) with hand grip strength and electrophysiological measures

| Age [yr], mean ± SD (range) | 37.3 ± 11.8 (18 – 57) |
| --- | --- |
| Sex, n (%) male  female | 11 (45.8)  13 (54.2) |
| SMA type, n (%) 2  3 | 9 (37.5)  15 (62.5) |
| SMN2 copy number, n (%) 2  3  4 | 2 (8.3)  13 (54.2)  9 (37.5) |
| Weight [kg], mean ± SD (range) n=24 | 58.4 ± 15.4 (27 - 90) |
| Height [cm], mean ± SD (range) n=24 | 163.9 ± 13.7 (140 - 187) |
| Mobility, n (%) ambulatory  non-ambulatory | 6 (25)  18 (75) |
| CK [U/L], mean ± SD (range)  n=24 | 183.63 ± 196.86 (15.6 – 687.6) |
| Crn [µmol/L], mean ± SD (range)  n=23 | 21.61 ± 13.92 (6.0 – 59.0) |
| HFMSE, mean ± SD (range)  n=24 | 14.8 ± 14.4 (0 - 41) |
| RULM, mean ± SD (range)  n=24 | 20.6 ± 11.1 (0 - 37) |
| ALSFRS-R, mean ± SD (range)  n=24 | 32.5 ± 5.8 (21 - 43) |
| 6MWT, mean (range)  n=5 | 194 (60 - 385) |
| Baseline hand grip strength [kg], mean ± SD (range)  n=23 | 4.8 ± 6.5 (0 – 27.6) |
| Baseline MUNIX APB, mean ± SD (range)  n=23 | 89.5 ± 62.7 (4 – 212) |
| Baseline MUSIX APB, mean ± SD (range)  n=23 | 125.3 ± 92.3 (56 – 484) |
| Baseline CMAP APB [µV], mean ± SD (range)  n=23 | 7738.5 ± 2945.9 (1837 – 13002) |

CK = serum creatine kinase activity; Crn = serum creatinine concentration; HFMSE = Hammersmith Functional Motor Scale Expanded (range 0-66); RULM = Revised Upper Limb Module (range 0-37); ALSFRS-R = revised ALS-Functional Rating Scale (range 0-48); 6MWT = Six Minute Walk Test; MUNIX = motor unit number index, MUSIX = motor unit size index; CMAP = compound muscle action potential; APB = abductor pollicis brevis muscle

**Suppl. Table S2** Correlations between CK / Crn and hand grip strength and electrophysiological measurement in nusinersen-naïve adult patients with SMA

|  | | CK [U/L] | Crn [µmol/L] |
| --- | --- | --- | --- |
| Hand grip strength (dominant hand) | *ρ* = | 0.694 | 0.586 |
|  | p value | <0.001 | <0.01 |
|  | n = | 23 | 23 |
| MUNIX APB | *ρ* = | 0.627 | 0.638 |
|  | p value | <0.01 | <0.01 |
|  | n = | 23 | 22 |
| MUSIX APB | *ρ* = | - 0.614 | - 0.697 |
|  | p value | <0.01 | <0.001 |
|  | n = | 23 | 22 |
| CMAP APB | *ρ* = | 0.580 | 0.290 |
|  | p value | <0.01 | n.s. |
|  | n = | 23 | 22 |

CK = serum creatine kinase activity; Crn = serum creatinine concentration; MUNIX = motor unit number index, MUSIX = motor unit size index; CMAP = compound muscle action potential; APB = abductor pollicis brevis muscle

**Suppl. Table S3** Changes in CK, Crn and HFMSE during the observation period 18 months after listwise exclusion of data using Friedman test with post-hoc Dunn-Bonferroni adjustment

|  |  | CK [U/L] | Crn [µmol/L] | HFMSE  (out of 66) |
| --- | --- | --- | --- | --- |
|  | n | 58 | 69 | 47 |
| Baseline | Mean ± SD | 306.64 ± 430.60 | 23.57 ± 16.83 | 23.7 ± 21.2 |
| 2-month analysis | Mean ± SD | 294.27 ± 446.11 | 23.94 ± 14.85 | 24.9 ± 21.7 |
|  | Mean difference versus baseline | - 12.37 (- 4.0%) | + 0.37 (+ 1.6%) | + 1.2 (+ 5.1%) |
|  | p value | n.s. | n.s. | n.s. |
| 6-month analysis | Mean ± SD | 290.73 ± 394.85 | 23.58 ± 14.16 | 25.2 ± 22.1 |
|  | Mean difference versus baseline | - 15.91 (- 5.2%) | + 0.01 (0%) | + 1.5 (+ 6.3%) |
|  | p value | n.s. | n.s. | n.s. |
| 10-month analysis | Mean ± SD | 284.08 ± 426.07 | 23.63 ± 14.47 | 25.9 ± 22.4 |
|  | Mean difference versus baseline | - 22.56 (- 7.4%) | + 0.06 (+ 0.3%) | + 2.2 (+ 9.3%) |
|  | p value | <0.01 | n.s. | <0.01 |
| 14-month analysis | Mean ± SD | 261.74 ± 347.19 | 24.40 ± 14.32 | 25.7 ± 22.4 |
|  | Mean difference versus baseline | - 44.90 (- 14.6%) | + 0.83 (+ 3.5%) | + 2.0 (+ 8.4%) |
|  | p value | <0.01 | n.s. | <0.01 |
| 18-month analysis | Mean ± SD | 256.04 ± 360.35 | 25.37 ± 14.44 | 25.9 ± 22.5 |
|  | Mean difference versus baseline | -50,6 (- 16.5%) | + 1.80 (+ 7.6%) | + 2.2 (+ 9.3%) |
|  | p value | <0.001 | <0.05 | <0.01 |

CK = serum creatine kinase activity; Crn = serum creatinine concentration; HFMSE = Hammersmith Functional Motor Score Expanded
